# 65-year-old Female with Cardiac Arrest and Return of Spontaneous Circulation

**DOI:** 10.5811/cpcem.2018.6.38420

**Published:** 2018-07-18

**Authors:** Megan Kirk, Leen Ablaihed, Zachary D.W. Dezman, Laura J. Bontempo

**Affiliations:** * University of Maryland Medical Center, Department of Emergency Medicine, Baltimore, Maryland; † University of Maryland School of Medicine, Department of Emergency Medicine, Baltimore, Maryland

## CASE PRESENTATION

A 65-year-old female was transported to the emergency department (ED) at approximately 2:00 AM following a witnessed cardiac arrest. According to the patient’s husband, she had been asleep in bed when she awoke suddenly, sat upright, and reached for her albuterol inhaler before “collapsing.” He found her to be pulseless and initiated cardiopulmonary resuscitation (CPR) while placing a call to emergency medical services (EMS). On EMS arrival, the patient was unresponsive and continued to receive CPR. She was intubated in the field using a size 7.0 endotracheal tube. Her initial rhythm was pulseless electrical activity (PEA), but she converted to normal sinus rhythm after receiving 1mg of epinephrine intravenously and 15 total minutes of CPR. No further history was available.

Per her husband, her past medical history was notable for “thyroid problems.” Her only medications were an albuterol inhaler, recently prescribed by her primary physician, and a multivitamin. She had no known drug allergies. On social history, the patient was not known to drink alcohol, smoke cigarettes, or use other substances. A family medical history and review of systems could not be obtained due to the acuity of her condition.

On examination, the patient was an obese female, intubated, and unresponsive. Her temperature was 37.1 degrees Celsius, blood pressure was 97/65 millimeters Hg, heart rate was 75 beats per minute (bpm). Her body mass index was estimated at 32. She was initially receiving assisted ventilation by EMS, but on examination in the ED she was found to have a spontaneous respiratory rate of 12 breaths per minute with an oxygen saturation of 98% on 40% fraction of inspired oxygen. Her head was atraumatic and normocephalic. Her pupillary exam showed mid-dilated symmetric pupils with sluggish reactivity to light. There was no hemotympanium or Battle’s sign. She had no apparent facial droop. An oral endotracheal tube was in place, confirmed with radiography and audible bilateral breath sounds. She had a full, supple neck without palpable masses, but additional exam was limited by body habitus. On cardiopulmonary exam, her lungs were clear to auscultation bilaterally and her heart had a regular rate and rhythm with no murmurs, gallops, or rubs. The patient’s abdomen was soft and nondistended with normal bowel sounds. On neurologic exam, her Glascow Coma Scale was 3T. She had diffusely decreased muscle tone, and 1+ patellar and brachioradialis deep tendon reflexes. Her musculoskeletal exam was unremarkable for deformity, erythema, or edema. Skin exam did not show any rashes, wounds or other lesions.

**Image 1:**
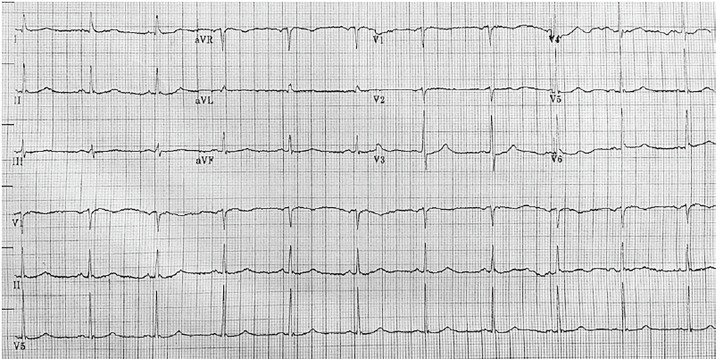
Electrocardiogram taken on the patient’s arrival to the emergency department.

**Image 2:**
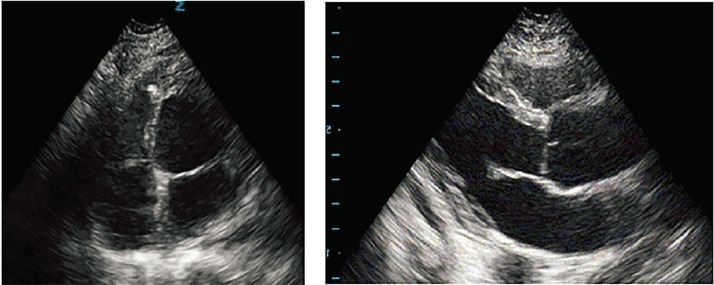
Point-of-care echocardiogram with an apical four-chamber view (left) and a parasternal long-axis view (right).

**Image 3:**
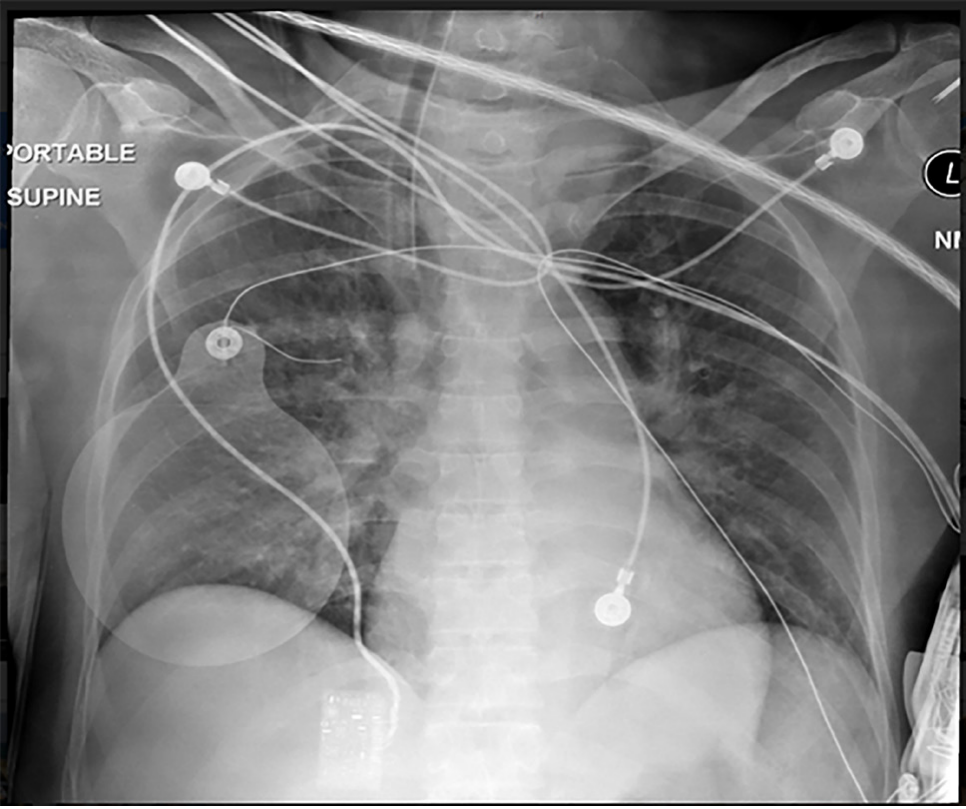
Anterior-posterior bedside chest radiograph.

Initial electrocardiogram (ECG) (Image 1) showed normal sinus rhythm at a rate of 70 bpm, normal axis, normal intervals and no pathologic t-wave inversions or ST-segment changes. A complete blood count and complete metabolic panel were done (Table 1). Additional laboratory tests, including thyroid studies, were unremarkable except for an elevated lactic acid of 6.9 millimoles/liter (L) (Table 2). A point-of-care echocardiogram was performed, which demonstrated grossly normal heart chamber sizes and systolic function with no pericardial effusion (Image 2). A point-of-care ultrasound of the abdomen and thorax was negative for any intra-abdominal free fluid. There was bilateral lung sliding present and no B lines. An anterior to posterior chest radiograph (CXR) is shown in Image 3.

The etiology of the patient’s cardiac arrest was unknown until a further diagnostic test was performed that revealed the diagnosis.

## CASE DISCUSSION

The presentation is that of a 65-year-old female who awoke with sudden shortness of breath then went into cardiac arrest, presumably with an initial rhythm of PEA. Given the history provided, a few things stood out.

Waking up with sudden dyspnea can be a symptom of many underlying diseases or conditions. Congestive heart failure is one of the more common causes of nocturnal dyspnea, but the patient had no symptoms or known diagnosis of previous heart failure. In fact she has a primary care doctor whom we know she had seen recently, had no complaints of limb swelling, and she did not have edema on exam when she presented to the ED. Her pro-brain natriuretic peptide was 96 picogram/milliliter and her CXR showed clear costal margins with no signs of pulmonary edema or effusions. Her point-of-care echocardiogram showed no B-lines, overt hypokinesis, or low ejection fraction. All of this points away from heart failure as the cause of her presentation.

Asthma, nocturnal asthma, and chronic obstructive pulmonary disease all cause nighttime wheezing and shortness of breath and respond to inhaled beta-agonists, such as the patient had at home. But this patient had no diagnosis of asthma, was not on chronic inhaled steroids, had no wheezing on exam, and no evidence of hyperinflation of her lungs on her CXR. Although her primary care physician recently prescribed her an albuterol metered-dose inhaler, adult onset asthma is rare and this patient has no known risk factors, except for obesity. Specifically, I am told that she never smoked and has no known allergies.

Pneumonia and upper respiratory infections (URI) with post-nasal drip can cause shortness of breath that might worsen at night. But this patient had no history of coughing or fever and the patient’s husband states her health was good. Psychiatric disorders such as anxiety or panic attacks can cause shortness of breath but there is nothing in the history to suggest these conditions, and in the ED these are diagnoses of exclusion. People with large body habitus, obstructive sleep apnea (OSA) or upper airway obstruction often complain of nighttime shortness of breath or wake up feeling suffocated. Although OSA was not mentioned in her history, this obese patient may simply not be diagnosed and this entity should remain on our differential.

Initial presentation in PEA arrest: When emergency providers hear the words PEA arrest, we are trained to go through all the “H’s and T’s” that cause PEA arrest. There was no history of bleeding, poor oral intake, or evidence to suggest hypovolemia on the patient’s lab results. Additionally, her point-of-care cardiac and focused assessment in shock and trauma ultrasound examinations did not show evidence of volume loss. Her labs also showed no evidence of hypokalemia, hyperkalemia or hypoglycemia. She was not hypothermic (temperature of 37.1° C). Hydrogen ion (acidosis) is evident by her gap acidosis of 20, which is explained by an elevated lactate of 6.9 mmol/L. Her hyperlactemia, however, may be secondary to her cardiac arrest and not the primary cause of her illness. Her delta gap (calculated as actual anion gap - 12 + bicarbonate [HCO_3_]) is 8. This is small and suggests an uncomplicated metabolic acidosis. Even after working through the MUDPILES mnemonic (Methanol, Uremia, Diabetic/alcoholic/starvation ketoacidosis, Paracetamol/phenformin/paraldehyde, Iron/isoniazid/inborn errors of metabolism, Lactic acidosis, Ethanol/ethylene glycol, salicylates), we return to lactic acidosis. Infection and sepsis are the common causes of hyperlactemia. She did not have any symptoms of infection prior to her arrest, and she doesn’t have any signs of overwhelming infection on exam. Her white blood cell count and vital signs are normal, aside from a borderline low blood pressure, and are not consistent with a diagnosis of sepsis. The lactic acidosis is therefore probably entirely due to her cardiac arrest and not an underlying condition.

Hypoxia cannot be excluded because we do not know her oxygen saturation when EMS arrived or after her pulses returned. An arterial blood gas would be helpful, but this was not a part of the case as presented. There was nothing in the history that would concern us for toxic substances ingested (see MUDPILES above). Her exam was inconsistent with a drug toxidrome, and she had no evidence of illicit drug use on her exam such as track marks. The patient had no history or signs of trauma. Looking at the point-of-care cardiac ultrasound, I saw no fluid in the pericardial space and therefore no pericardial tamponade. I also did not see any signs of right heart strain as the right ventricle was grossly normal in size and the intraventricular septum was not bowed, making a massive pulmonary embolism unlikely. Although it is difficult to see wall motion abnormalities on a point-of-care ultrasound, it was reported that the patient’s ejection fraction was only minimally depressed. The patient’s ECG showed no evidence of ST-segment elevation or depression, and her initial troponin was not elevated, thereby making an acute myocardial infarction (MI) as the primary cause of the patient’s presentation unlikely. Thus, thrombus (both coronary and pulmonary) is unlikely to be the cause of this patient’s arrest. Tension pneumothorax was excluded by looking at her CXR. After going through the list of “H’s and T’s,” hypoxia and hydrogen ions remain on the differential of what might have caused her arrest.

The PEA arrest differential can also be divided into etiologies that cause a narrow complex vs. a wide complex rhythm.^1^ I was not told if the patient’s rhythm upon EMS arrival was narrow or wide. However, using the same logic applied to the “Hs and T’s,” the common causes of narrow complex PEA (cardiac tamponade, tension pneumothorax, mechanical hyperventilation, pulmonary embolism and acute MI) have already been eliminated. Wide complex etiologies such as hyperkalemia and acute MI have likewise been excluded. An additional cause of wide complex PEA, sodium channel blocker toxicity, has not been disproven but there is nothing in the history to suggest this as the cause. The patient’s only reported medication is an albuterol inhaler and there is no known history of cocaine use.

She had return of spontaneous sinus rhythm after EMS intervened. Was the cause of her PEA arrest reversed by one of the interventions provided by EMS? PEA is generally due to a non-cardiac cause, and by addressing the underlying cause a patient can regain his/her pulse. EMS intubated the patient and gave her epinephrine. For the latter, epinephrine is a short-lived drug and its effects should have worn off by the time she reached the ED. For the former, the patient could have been in cardiac arrest due to respiratory failure, which was reversed once she was intubated. A third, and unlikely, possibility is that she was never in arrest at all but had difficult-to-palpate pulses and was in pseudo-PEA.

Given her potential causes of hypoxia, including OSA and upper airway obstruction, we turn to the CXR. There were no signs of pneumothorax or subcutaneous emphysema. There are no lobar infiltrates suggestive of a pneumonia or other intraparenchymal pathology, no signs of hyperinflation, no signs of pulmonary edema (“bat wings”), and no signs of pleural effusions. Looking at her trachea and the placement of the endotracheal tube, we can see it is clearly deviated to the right. There is a large soft-tissue mass in the left side of the neck that extends into the thoracic inlet causing the trachea to shift to the right. This finding made me very concerned for an upper airway mass that compressed her trachea. This could worsen when the patient is supine and sleeping and could have caused the patient’s cardiac arrest. This would be reversed by establishing an airway with a rigid endotracheal tube by EMS. Here’s the question: What is the etiology of the mass causing this radiograph finding?

The differential diagnosis of tracheal deviation is divided into the following:

- Pulmonary causes:
  ○ Deviated towards the diseased side: atelectasis, agenesis of the lung, pneumonectomy, and pulmonary fibrosis
  ○ Deviated away from the diseased side: pneumothorax, lung mass, pleural effusion
  ○ All of these conditions are unlikely given the imaging provided.
- Other causes:
  ○ Retrosternal goiter, mediastinal masses, tracheal masses, thyroid cancer, and kyphoscoliosis.

Putting all the information together, we have an obese female who presented after a PEA arrest. The arrest appears to have been due to hypoxic respiratory failure and was reversed by intubation. Her history was notable for intermittent shortness of breath for months and a “thyroid problem.” Her medical history, exam, and ED workup did not support a cardiac or pulmonary condition as the primary cause of her presentation. She did have an elevated lactate, but the other information provided in the case does not support a diagnosis of sepsis. Her CXR reveals a neck mass with tracheal deviation. Neck masses generally originate from the thyroid, yet this patient had normal thyroid hormone levels. Some causes of euthyroid masses include thyroid cancer, uni- or multinodular goiter, diffuse goiter, autoimmune thyroiditis, de Quervain’s thyroiditis, and Riedel’s thyroiditis.

The question I now asked myself was this: Can a large goiter or thyroid mass have an initial presentation of hypoxia and upper airway obstruction? The answer, after a quick review of the literature, was, “Yes!” There are many case reports in the literature that discuss similar presentations of patients presenting with sudden extreme upper airway obstruction requiring emergent intubation.^2^–^8^ Therefore, the test of choice would be a computed tomography (CT) of the neck and chest revealing what I expect would be a goiter causing tracheal deviation and obstruction.

## CASE OUTCOME

**Image 4:**
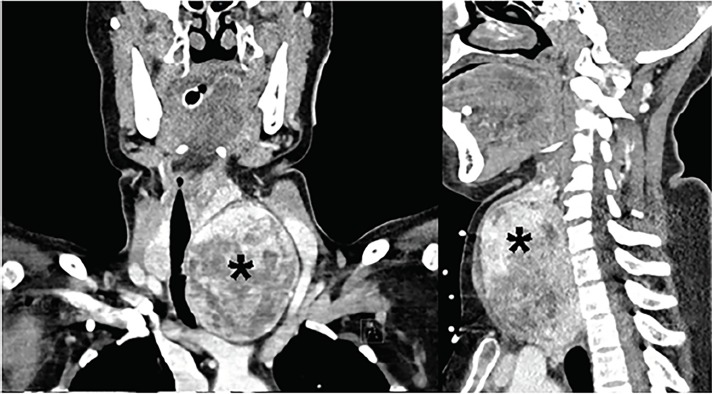
Computed tomography of neck and soft tissues showing a large substernal goiter (*) with tracheal compression (frontal plane on left, sagittal plane on right).

The diagnostic test ordered was a CT of the soft tissues of the neck, which demonstrated a large substernal thyroid goiter with significant tracheal compression (Image 4). The patient had an overall unremarkable physical exam and laboratory findings due to EMS stenting open the obstructed trachea by placing an endotracheal tube in the field. The profound rightward tracheal deviation on CXR indicated a space-occupying lesion in the neck and chest, prompting further imaging of the neck. Ultimately, the patient was found to have a severe degree of hypoxic encephalopathy. She was removed from ventilatory support and expired on her fourth hospital day. Later chart review revealed she had been diagnosed with a multinodular thyroid goiter 10 years ago but had declined any workup and had been lost to follow-up. Her recent prescription of an albuterol inhaler suggests she had been experiencing shortness of breath but did not attribute it to her large goiter.

## RESIDENT DISCUSSION

Thyroid goiter is a common problem worldwide, with iodine deficiency being responsible for the plurality of cases. Within the United States and other “iodine-replete” countries, multinodular goiter, chronic autoimmune thyroiditis (Hashimoto’s), and Grave’s disease are the more likely etiologies.^9^ Multinodular goiter, the cause of our patient’s pathology, is generally due to an unclear genetic predisposition and worsens with age. Chronic autoimmune thyroiditis is caused by excessive stimulation of the thyroid gland by thyroid-stimulating hormone (TSH). Grave’s disease is attributed to over-activation of the TSH receptor by antibodies. Women are much more likely than men to develop a thyroid goiter.^9^–^12^

Approximately 10–20% of goiters are ultimately malignant in nature regardless of location.^9^,^10^ Few cause tracheal compression or obstruction. Large, rapidly growing, or posteriorly-positioned goiters are more likely to result in obstructive symptoms. Historical factors worrisome for progressive obstruction include longstanding goiter, cough, dysphagia, hoarse voice, or superior vena cava syndrome (SVCS).^11^–^13^ Exertional dyspnea is frequently experienced when tracheal diameter is compressed to less than eight millimeters, and stridor manifests when the lumen is less than five millimeters.^14^ Positional changes, such as reaching forward, bending at the waist and lying supine, can provoke symptoms of obstruction.^13^

Compressive thyroid goiters are generally managed surgically on a non-emergent basis, although our case demonstrates they can cause complete airway loss.^14^,^15^ Partial airway obstruction can rapidly progress to complete obstruction if there is an additional insult, including even mild edema from an URI. Thyroid tissue is also especially vascular and nodules are prone to expansion if bleeding occurs within the gland, which can rapidly increase the size of a previously slow-growing goiter.^14^ Emergent airway management is the primary treatment in all of these scenarios. Passage of an orotracheal tube is ideal for such patients (as performed by EMS in our case), but generally a somewhat smaller caliber tube is required than what would be expected for an adult of a given size.^14^,^16^ Caution should be used when considering an emergency surgical airway (cricothyrotomy) as these patients are likely to have marked tracheal deviation and an overlying vascular mass complicating the procedure.

Complete tracheal compression resulting in a (likely hypoxic) PEA arrest is an uncommon presentation of a common illness (thyroid goiter), but demonstrates that compressive mediastinal masses can drastically impact a patient’s health. Beyond the thyroid, other origins of compressive mediastinal mass to consider in such a patient are thymoma, teratoma, lymphoma, and the aorta.

## FINAL DIAGNOSIS

Hypoxic cardiac arrest secondary to tracheal compression from substernal thyroid goiter.

## KEY TEACHING POINTS

- Thyroid goiter is more common in women and in the U.S., it is most frequently attributed to multinodular goiter, Grave’s disease, or chronic autoimmune thyroiditis.
- Laboratory testing is largely unhelpful in euthyroid goiter.
- When goiters develop substernally they can cause significant tracheal compression plus the following:
  ○ Dyspnea on exertion
  ○ Cough
  ○ Dysphagia
  ○ Dysphonia
  ○ Stridor
  ○ SVCS
  ○ Tracheal deviation/luminal narrowing
  ○ Respiratory arrest (rarely)
- Management is generally surgical excision.
- Endotracheal intubation instead of cricothyrotomy is the preferred means of airway management in these patients.

Documented patient informed consent and/or Institutional Review Board approval has been obtained and filed for publication of this case report.

## Figures and Tables

**Table 1:** Hematology and chemistry studies.

Hemoglobin	11.7 g/dL
Hematocrit	36.5%
Sodium	140 mmol/L
Chloride	100 mmol/L
Potassium	3.0 mmol/L
Bicarbonate	20 mmol/L
White blood count	7.1 K/mcL
Platelets	193 K/mcL
Blood urea nitrogen	14 mg/dL
Creatinine	1.0 mg/dL
Glucose	291 mg/dL

*G*, grams; *dL*, deciliter; *mcL*, microliter; *mmol*, millimoles; *L*, liter; *mg*, milligrams.

**Table 2:** Additional laboratory values.

Total protein	6.3 g/dL
Albumin	3.6 g/dL
Direct bilirubin	<0.2 mg/dL
Total bilirubin	0.2 mg/dL
Aspartate aminotransferase	39 u/L
Alanine aminotransferase	25 u/L
Alkaline phosphatase	73 u/L
Lipase	46 u/L
Pro-brain natriuretic peptide	96 pg/mL
Thyroid stimulating hormone	1.18 u/mL
Troponin I	< 0.01 ng/mL
Lactate	6.9 mmol/L

*G*, grams; *dL*, deciliter; *mg*, milligrams; *u*, units; *L*, liter; *pg*, picograms; *mL*, milliliters; *ng*, nanograms; *mmol*, millimoles.
